# Evaluation of the Bruker SMART X2S: crystallography for the nonspecialist?

**DOI:** 10.1107/S0021889810042561

**Published:** 2010-11-27

**Authors:** Kevin S. Eccles, Stephen P. Stokes, Carla A. Daly, Nicola M. Barry, Sharon P. McSweeney, Damian J. O’Neill, Dawn M. Kelly, W. Brian Jennings, O. M. Ní Dhubhghaill, Humphrey A. Moynihan, Anita R. Maguire, Simon E. Lawrence

**Affiliations:** a Department of Chemistry, Analytical and Biological Chemistry Research Facility, University College Cork, Ireland; b Department of Chemistry and School of Pharmacy, Analytical and Biological Chemistry Research Facility, University College Cork, Ireland

**Keywords:** Bruker SMART X2S, instrumentation, automation

## Abstract

An evaluation of the Bruker SMART X2S for the collection of crystallographic diffraction data, structure solution and refinement is carried out with a variety of materials with different electron densities, presenting some of the successes and challenges of automation in chemical crystallography.

## 1. Introduction

Chemical crystallography is a mature science in which structural analysis of well formed single crystals is routine for many samples (Ooi, 2010 ▶), with the largest amount of time spent on problematic cases, such as twinning, disorder *etc.* (Herbst-Irmer & Sheldrick, 1998 ▶; Müller, 2007 ▶, 2009 ▶). Automation is developing for both chemical and biological crystallography (Adams *et al.*, 2010 ▶; Dolomanov *et al.*, 2009 ▶; Fuller *et al.*, 2010 ▶). Automation can increase awareness of a technique, but can also lead to reduced understanding and knowledge of the scientific theory involved and reduced appreciation of its difficulties or limitations. A criticism is that it leads to a ‘black box’ philosophy, characterized by noncritical appraisal of, and over-reliance on, the results obtained.

The Bruker SMART X2S is a benchtop crystallography instrument designed to enable more widespread use of crystallography in the wider chemical community, in the same way that NMR and mass spectrometry have become commonplace. The design has centred not only on automatic data collection, structure solution and refinement, but also on some critical analysis of the structural results obtained. The main features are the air-cooled Breeze CCD detector and an Mo microfocus source. The use of CCD detectors for X-ray diffraction is a mature technology (Gruner *et al.*, 2002 ▶), with an air-cooled detector available since 2006. [See supplementary information^1^ for further details of the instrument; see also Kirschbaum *et al.* (1997 ▶) and Schulz *et al.* (2009 ▶).] Herein, we discuss our experiences with the Bruker SMART X2S, presenting data for a representative range of chemical samples, highlighting its successes and challenges.

## 2. Sample preparation – alignment

Correct sample alignment remains critical for good quality diffraction data (Müller, 2009 ▶) and is a major consideration for any automated process. The effect of sample misalignment on the overall data quality and success of the instrument has been investigated with a crystal (0.24 × 0.28 × 0.29 mm) of dibenzyl sulfone, (1), for which a crystal structure had been previously reported by Rudolph *et al.* (2010 ▶). Three experiments were run with the crystal intentionally placed in the following positions: (i) correctly in the middle of the mount, (ii) incorrectly below the centre of the mount and (iii) incorrectly to the side of the centre of the mount. Table 1 in the supplementary information summarizes the data.

The first two experiments have similar data, both of which are perfectly acceptable for publication: a slight increase in *R*1, *wR*2 and goodness of fit (GooF) for the second experiment suggests the overall data quality is slightly worse, and the experiment took longer. For the third experiment, the crystal is sufficiently far from the centre of the mount that it is precessing in and out of the beam. Thus, the symmetry-equivalent reflections do not match, the Laue check fails and the larger centred unit cell is not identified. The checkCIF output highlights the missed symmetry, as well as the high *R*_int_ and final *R* values, and should alert the nonspecialist to the fact that there is a problem.

In summary, the large beam size means that alignment is not as critically important as on other instruments, particularly in the horizontal direction, although for short data collection times and good quality data, it is still important.

## 3. Sample preparation – sample size

Crystal size is of paramount importance for successful experiments (Müller, 2009 ▶). Different sized crystals of *N*-cyano-*S*-benzyl-*S*-(2-fluorophenyl)sulfilimine, (2), synthesized (Barry *et al.*, 2009 ▶) and obtained from the same batch, were used to investigate the effects of crystal size on the capabilities of the SMART X2S. The effective minimum crystal size limit was of particular interest. Table 2 in the supplementary information summarizes the results, which show that the minimum practical crystal dimensions for a moderate scatterer, in this case S, are 0.20 mm in two directions and 0.1 mm in the third. For smaller dimensions, the system aborted data collection owing to insufficient diffraction from this compound.

Of course, one cannot completely generalize from these results to all samples since the diffracting power of each compound depends upon a number of different factors, *e.g.* the scattering power of the atoms, the degree of disorder, the crystal mosaicity *etc.*

## 4. Correct structure assignment

The reliability of the software for a range of compounds to which we have ready access was tested, *viz*. transition metal complexes, organic compounds, cocrystals and hydrates. Compounds (3), (4) and (11) are novel (see the supplementary information for their synthesis). Literature methods were used for the synthesis of (5) (Feng *et al.*, 2009 ▶), (6) (Singh *et al.*, 2002 ▶), (7) (Barbosa *et al.*, 2009 ▶), (8) (Takada *et al.*, 1997 ▶) and (9) (Brondel *et al.*, 2010 ▶). Compound (10) was obtained from Sigma–Aldrich. The crystal structures of (9) and (10) (Himes *et al.*, 1981 ▶) are known. For all compounds the correct structure was obtained, with satisfactory results in terms of *R* factor, GooF, C—C bond precision *etc.* (see supplementary information, Scheme 1 and Table 3). The polarity of compound (9), as evidenced by the Flack (1983 ▶) parameter, was also correctly assigned. Thus, for these compounds, the hardware and software work well to produce crystallographic and structural data of publishable quality, suitable for deposition in the Cambridge Structural Database (Allen, 2002 ▶).

## 5. Incorrect structure assignment – one-electron differences

Scheme 2 and Table 4 in the supplementary information show that for compounds (12)–(14) an incorrect structure was obtained. The data quality seems fine, with no evidence of twinning or disorder, so what types of issues have occurred? The errors involve differentiation between atoms that differ by one electron, *e.g.* N and O atoms are reversed in (12) (Wardell *et al.*, 2005 ▶), and C and N in (13) (Kiran *et al.*, 2008 ▶; Kelly *et al.*, 2010 ▶). For (14) (synthesis previously described; Barry *et al.*, 2009 ▶), an extra H atom has been placed on the N atom attached to the S atom, which is part of the unusual functional group S=N—C≡N. This group was also found in (2), for which there were no problems. Interestingly, the crystal is a racemic twin, although whether this has caused the incorrect assignment is unclear. The software did detect that there was a problem, which it attributed to twinning, and finished at this point.

Scientists have always scrutinized crystallographic results to verify that the structure makes chemical sense. This is still important, no matter what combination of hardware and software is producing the crystallographic result. As with all crystallographic experiments, evidence from other techniques is always required.

## 6. Crystals with multiple moieties

Compounds (2)–(14) are anhydrous samples, without any solvent present. The effectiveness of the system for crystals containing more than one compound was investigated: a hydrate, a solvate and a cocrystal, (15)–(17) (see the supplementary information for their synthesis; see also Scheme 3 and Table 5).

The structures of both (15) and (17) were assigned correctly. For (16), a C and an N atom were misassigned, as discussed in §[Sec sec5]5. The H atoms of the water molecule were not assigned, probably because of the low scattering ability of hydrogen and the fact that the experiment was not performed at low temperature. Interestingly, this is a new polymorph of (16), which has been prepared by a different method to the known polymorph (Alléaume *et al.*, 1976 ▶) and has been confirmed by powder X-ray diffraction analysis of bulk samples of both polymorphs, as well as a comparative data collection on a Bruker APEX DUO (see §[Sec sec8]8).

## 7. Inputting the incorrect formula

The effect of inputting the incorrect molecular formula was investigated, since it is necessary to input a formula at the start of the experiment, and in some cases the identity of the crystal may not be known. For example, (5), C_13_H_10_OS, was incorrectly input as the sulfoxide, C_13_H_10_O_2_S, and (10), C_15_H_12_N_2_O, was input as C_27_H_22_N_2_O_2_S. The hydrate (16) and solvate (15) were input as the pure material. The software is robust and coped with an incorrect molecular formula in the majority of cases, with 80% of samples obtaining the correct structure.

There is one issue that causes inconvenience. The system does not update the CIF and report files with the molecular formula based on the structure obtained, but instead uses the formula input by the user. This requires manual refinement for cases where the submitted formula is different from the structure obtained.

## 8. Comparison with a Bruker APEX DUO

A comparative study with a Bruker APEX DUO was undertaken at room temperature using a sealed-tube Mo *K*α source for (2), (8), (11), (12), (16) and (18), which include samples of both good and poor crystal quality. The synthesis of (18), 4-methyl-*N*-phenylbenzenesulfonamide, has been described by Massah *et al.* (2006 ▶). The results, summarized in Tables 6 and 7 in the supplementary information, are comparable for the two instruments. The biggest difference is the higher intensity of the incident beam of the APEX DUO, leading to shorter experiments for poor quality crystals.

## 9. Concluding remarks

The SMART X2S is a benchtop instrument designed for routine chemical crystallography and powered from a normal mains supply. The combination of the Breeze detector and the Mo microfocus source means that good quality data from crystals of large to moderate size can be collected, solved and refined without external help within a few hours. For all our samples the overall success rate is approximately >90% for correct structure assignment, rising to >99% when off-line refinement has been undertaken. (There have been two samples from over 200 experiments for which data collection has taken place and we have been unable to solve the structure.)

The checkCIF output allows fast diagnosis of any issues in the experiment. Inputting an incorrect formula at the start of the experiment, for example, will immediately become obvious from the checkCIF output because of differences in formula, density *etc*. In our experience, those users who are familiar with the checkCIF output after an experienced crystallographer has finalized a crystal structure are asking more questions about the checkCIF output they obtain from the SMART X2S. Novice users are also asking similar questions and some of these questions are about the technique itself. This is a major advantage of the output from the instrument, in that it does seem to be increasing awareness of crystallography among the synthetic chemists.

Instrumentation at an early stage of evolution will inevitably present minor issues that are not optimal, or at least not to an end-user’s liking; for example, re-numbering of atoms has to be done off-line, using the *APEX2* software suite (Bruker, 2007 ▶) which is supplied with the SMART X2S. In addition, some extra cycles of refinement would be beneficial since the software does terminate too early in some cases, as evidenced by the Δ/σ values.

In some experiments, *F*_obs_ for the very low angle reflections are much smaller than *F*_calc_, with Δ*F* ^2^/σ values significantly higher (>10) than the rest of the data set (<5), owing to the beam stop blocking or partially blocking the correct measurement of these reflections in some orientations. This is not unusual for chemical crystallography and omitting these from the latter cycles of refinement would be useful, although it may be better not to do this in a routine manner.

In summary, the SMART X2S as an instrument has allowed chemists with no crystallography experience to obtain crystallographic data for novel compounds. The instrument has greatly increased the use of crystallography in the department, with little training required to operate a user-friendly and easy-to-use instrument.

The CIF data (Hall & McMahon, 2006 ▶) for all experiments are provided as supplementary information and have been deposited with the Cambridge Crystallographic Data Centre (CCDC) for the novel crystals (2)–(8), (11) and (14)–(18). [The following computer programs were used in the refinement: *APEX2*, *GIS*, *SADABS* and *SAINT* (Bruker (2009 ▶), *SHELXS97* and *SHELXL97* (Sheldrick, 2008 ▶), and *PLATON* (Spek, 2009 ▶).]

## Supplementary Material

Crystal structure: contains datablocks Compound_1_Below, Compound_1_Centre, Compound_1_Side, Compound_2_APEX, Compound_2_CCDC, Compound_2_Run_1, Compound_2_Run_2, Compound_2_Run_3, Compound_2_Run_4, Compound_3_CCDC, Compound_3_X2S, Compound_4_CCDC, Compound_4_X2S, Compound_5_CCDC, Compound_5_X2S, Compound_6_CCDC, Compound_6_X2S, Compound_7_CCDC, Compound_7_X2S, Compound_8_APEX, Compound_8_CCDC, Compound_8_X2S, Compound_9_X2S, Compound_10_X2S, Compound_11_APEX, Compound_11_CCDC, Compound_11_X2S, Compound_12_APEX, Compound_12_Manual, Compound_12_X2S, Compound_13_Manual, Compound_13_X2S, Compound_14_CCDC, Compound_14_Manual, Compound_14_X2S, Compound_15_CCDC, Compound_15_Manual, Compound_15_X2S, Compound_16_APEX, Compound_16_CCDC, Compound_16_Manual, Compound_16_X2S, Compound_17_CCDC, Compound_17_X2S, Compound_18_APEX, Compound_18_CCDC, Compound_18_Manual, Compound_18_X2S, global. DOI: 10.1107/S0021889810042561/kk5074sup1.cif

Structure factors: contains datablocks 2_CCDC. DOI: 10.1107/S0021889810042561/kk50742_CCDCsup2.hkl

Structure factors: contains datablocks 3_CCDC. DOI: 10.1107/S0021889810042561/kk50743_CCDCsup3.hkl

Structure factors: contains datablocks 4_CCDC. DOI: 10.1107/S0021889810042561/kk50744_CCDCsup4.hkl

Structure factors: contains datablocks 5_CCDC. DOI: 10.1107/S0021889810042561/kk50745_CCDCsup5.hkl

Structure factors: contains datablocks 6_CCDC. DOI: 10.1107/S0021889810042561/kk50746_CCDCsup6.hkl

Structure factors: contains datablocks 7_CCDC. DOI: 10.1107/S0021889810042561/kk50747_CCDCsup7.hkl

Structure factors: contains datablocks 8_CCDC. DOI: 10.1107/S0021889810042561/kk50748_CCDCsup8.hkl

Structure factors: contains datablocks 11_CCDC. DOI: 10.1107/S0021889810042561/kk507411_CCDCsup9.hkl

Structure factors: contains datablocks 14_CCDC. DOI: 10.1107/S0021889810042561/kk507414_CCDCsup10.hkl

Structure factors: contains datablocks 15_CCDC. DOI: 10.1107/S0021889810042561/kk507415_CCDCsup11.hkl

Structure factors: contains datablocks 16_CCDC. DOI: 10.1107/S0021889810042561/kk507416_CCDCsup12.hkl

Structure factors: contains datablocks 17_CCDC. DOI: 10.1107/S0021889810042561/kk507417_CCDCsup13.hkl

Structure factors: contains datablocks 18_CCDC. DOI: 10.1107/S0021889810042561/kk507418_CCDCsup14.hkl

Supplementary material file. DOI: 10.1107/S0021889810042561/kk5074sup15.pdf
Figures, tables and supplementary information

## Footnotes

1 Supplementary material for this paper is available from the IUCr electronic archives (Reference: KK5074). Services for accessing this material are described at the back of the journal.
