# Supplementary material for: Evaluation of the Bruker SMART X2S: crystallography for the nonspecialist?
Source: J Appl Crystallogr. 2010 Nov 27;44(Pt 1):213–5. doi: 10.1107/S0021889810042561 (PMC3253739; doi:10.1107/S0021889810042561)
Supplement: Supplementary file 15 [file j-44-00213-sup15.pdf]

Supplementary Information

1. Instrumentation and Software

The instrument consists of a fixed air-cooled micro-focus sealed Mo-Kα (0.71073 Å) X-ray source co-mounted with a doubly curved Si crystal monochromator and a fixed air-cooled Breeze CCD detector, Figure 1, which is housed in a 64 cm x 94 cm x 48 cm box weighing approx. 150 kg, using mains power from a standard plug.

There is no requirement for either cooling water or a large electricity supply, and the instrument requires low maintenance. In addition, there is a port for a low temperature device, if desired. The detector is at a fixed distance of 39.3 mm from the sample, at 2θ = -20.0 ° and the goniometer is fixed with χ = 35.1 º. The system is free to move in ω and ϕ. The instrument collects the data by three ω scans with a frame width of 0.5 º, the first is a 180 º scan from ω = -20 º to -200 º, with ϕ = 0 º. The second and third ω scans also start at ω = -20 º and the scans are 180 º / 120 º / 60 º, depending on the symmetry of the crystal, with ϕ = 120 º for the 2nd scan and ϕ = 240 º for the third scan. The resolution limit of the instrument is 0.84 Å. The beam is slightly elliptical in nature, with a wider profile in the horizontal direction, to allow for a degree of misalignment, as discussed later in Section 3. The beamstop is directly attached to the detector face, which allows for ready visualization of the source since it filters out most, but not all, of the direct beam. Figure 2 shows a representative diffraction pattern, in this case for compound **2**, discussed later in Section 4.

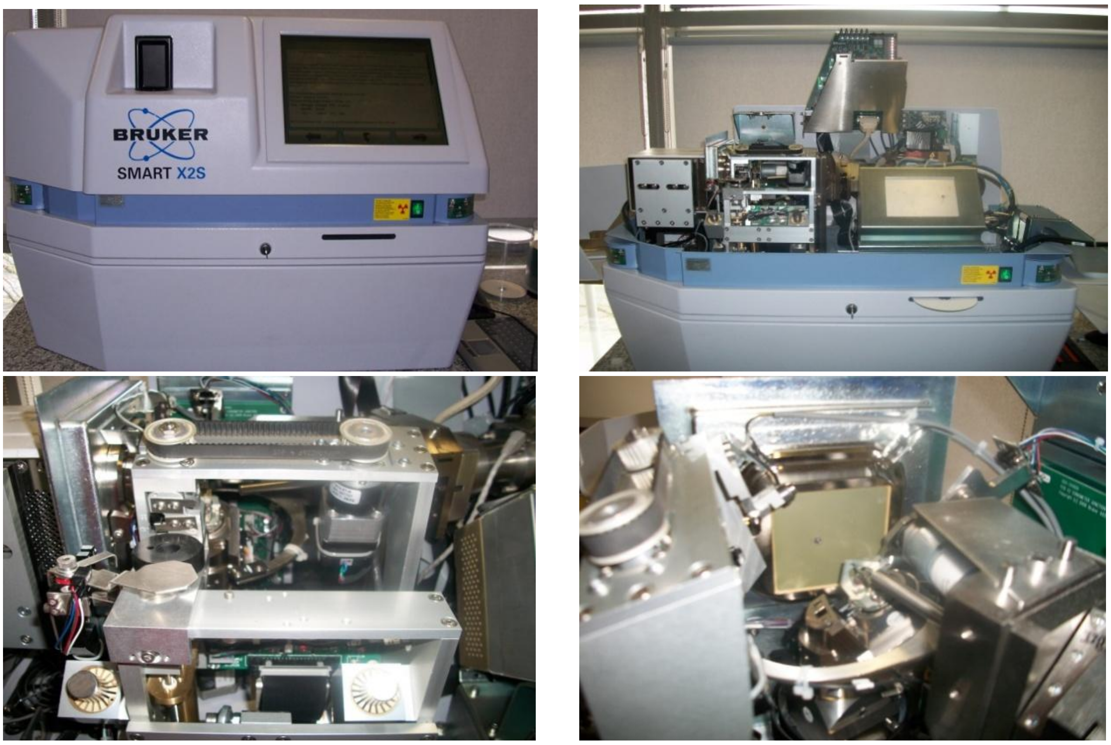

Figure 1: The Benchtop Bruker SMART X2S: external view (top left), cover removed (top right), patented sample loader (bottom left) and detector with beam stop (bottom right).

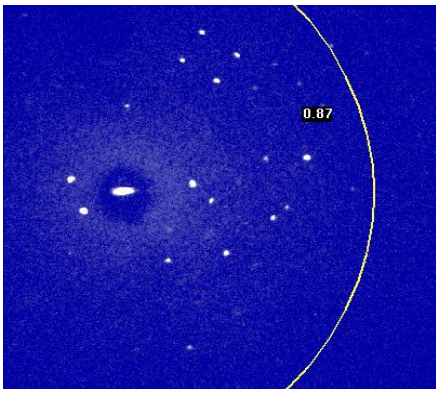

Figure 2: An example of the diffraction pattern obtained for compound **2**, sample 2, as discussed in Section 4. The direct beam is the large elliptical white area to the left-hand side of the image.

Samples are placed on pre-aligned Mitegen™ micromounts fitted on non-magnetic SPINE-type pins (0.64 mm) to a magnetic base. Samples are glued in place with UV curable glue. Once covered with a plastic top, this is placed in the loading port and transferred on to the goniometer via the sample loader. Note that the plastic top is removed and stored during the sample loading process. A CD is placed in the CD drive of the instrument and the Bruker Kiosk interface software (Bruker, 2009) allows information about the crystal size, habit and color, as well as expected molecular formula to be input in to the software.

From this point, the system operates in a fully automated fashion. An initial unit-cell is determined (Bruker, 2009), followed by data collection (Bruker, 2009), structure solution (Sheldrick, 2008) and refinement (Sheldrick, 2008) using the Autostructure™ software. This data, along with the complete dataset and CIF file (Hall *et al.* 2006) are written to the CD for publication or further work, as required. The data is also presented in various formats, including html with JMol for viewing the chemical structure. Note that manual refinement is required for non-routine samples which exhibit issues such as twinning or disorder. Both the sample, with plastic top, and CD are ejected at the end of the experiment.

Initially, the instrument collects three runs of twelve frames each for the unit-cell determination at 10 seconds exposure per frame. The system aborts if insufficient diffraction is observed after the first run. In cases where diffraction is seen but the unit-cell cannot be determined, the software calculates how many additional frames have to be collected until an appropriate number of reflections are available for cell refinement. If a unit-cell is determined, data collection proceeds at exposure times between 5 and 60 seconds per frame, depending on the quality of the data obtained for the unit-cell. Thus, the minimum crystal size that can be analysed is dependent on the successful determination of the unit-cell at exposure times of 10 seconds per frame.

For samples where diffraction is observed but the unit-cell cannot be determined, the instrument collects the most data possible, i.e. a full sphere of data, by undertaking three ω scans of 180 °. This is available for manual processing off-line and is seen for twinned crystals, for example, where the system knows it cannot use the indexing solution to solve the

structure. If the unit-cell does not give a clear-cut indication of the likely symmetry of the crystal, a dataset corresponding to the lower symmetry is collected.

After the first ω scan of 180 ° the software performs a Laue check and then re-refines the unit cell in the correct crystal system. It is at this point that the unit-cell displayed on the screen can change during data collection, since more information is now available compared to that used to determine the initial unit-cell initially. The information from the Laue check is used to determine the data collection strategy for the last two runs, for example changing the size of the ω scans.

The data collection time is chosen to acquire data with $I/\sigma(I)$ of 1.2 at the 0.84 Å limit. This is reflected in the resolution limit exhibited on screen during data collection, which can be lower for crystals which diffract poorly. In such cases, only the initial integration of the first run is done at 0.84 Å. The subsequent full integration of all runs is performed at the resolution determined from the first integration. After data collection, structure solution and refinement are undertaken using the entire dataset.

For experiments requiring manual off-line processing, this can be performed using the full functionality of the APEX2 software suite, which is supplied with the SMART X2S.

All experiments are stored on the internal hard drive, with the date and time noted, as well as written to CD, which allows the administrator to monitor usage, for charging purposes etc. There is also a new option, which has just been announced in March 2010, which enables multiple users to pre-schedule the SMART X2S instrument time.

2. General Procedures

All solvents used were HPLC grade or were distilled prior to use by the following methods: methylene chloride (CH_2Cl_2) was distilled from phosphorous pentoxide, ethyl acetate was distilled from potassium carbonate, hexane was distilled prior to use. Organic phases were dried using anhydrous magnesium sulfate ($MgSO_4$). All commercial reagents were supplied by Sigma-Aldrich. All commercial reagents were used without further purification.

^{1}H (300 MHz) and ^{13}C (75.5 MHz) NMR spectra were recorded on a Brüker AVANCE 300 NMR spectrometer. ^{1}H (400 MHz) and ^{13}C (100.0 MHz) NMR spectra were recorded on a Brüker AVANCE 400 NMR spectrometer. All spectra were recorded at 20 °C in deuterated chloroform ($CDCl_3$) or deuterated dimethyl sulfoxide (d_6-DMSO). Chemical shifts (δ_H and δ_C) are reported in parts per million (ppm) relative to the TMS signal and coupling constants are expressed in Hertz (Hz). Splitting patterns for ^{1}H NMR and ^{13}C NMR spectra are designated as follows: singlet (s), broad singlet (bs), doublet (d), doublet of doublets (dd), doublet of doublets of doublets (ddd), triplet (t), doublet of triplets (dt), quartet (q), AB quartet (ABq), multiplet (m).

Infra-red spectra were recorded as potassium bromide (KBr) discs or as thin films on NaCl plates on a Perkin Elemer Paragon 1000 FT–IR spectrometer in the range 4,000 – 600 cm^{-1}. Melting Point were measured on an Electrothermal 9100-melting point apparatus.

Microanalysis was performed by the Microanalysis Laboratory, UCC, Cork on Perkin-Elmer 240 and Exeter Analytical CE440 elemental analysers. Mass spectrometry was carried out using a Waters/Micromass Quattro Micro triplet quadrupole spectrometer (ESI) or a Kratos-profile HV-4 double focussing high resolution mass spectrometer (EI).

Synthesis of precursor to 3

A mixture of *iso*-butylamine (1.00 g, 1.36 mL, 13.65 mmol), 2-benzoylpyridine (0.50 g, 2.73 mmol), titanium(IV) ethoxide (2.86 mL, 3.11 g, 13.65 mmol) and 10 mL of CH_2Cl_2 was heated at reflux overnight while stirring. The reaction was cooled to room temperature and deionised water (5 mL) was added slowly. The solution was filtered through celite to remove traces of solid material, and washed with CH_2Cl_2 (3 x 5 mL). The organic layer was separated and the aqueous layer was extracted with CH_2Cl_2 (3 x 5 mL). The combined organic layers were washed with brine (5 mL), dried ($MgSO_4$), filtered and concentrated to yield a colourless oil (0.53 g, 81%). ^{1}H and ^{13}C NMR analysis showed that the reaction had gone to completion and the pure imine was obtained as E/Z isomers in a ratio of 1.0:1.3; ν_{max} (NaCl)/cm^{-1} 1632 (C=N), 1583 (C=N, pyridine ring); δ_H (300 MHz; $CDCl_3$) Major isomer: 0.94 (6H, d, $^3J_{H\text{-}H}$ 6.6, $C_{13}H_3$ + $C_{14}H_3$), 1.98 - 2.16 (1H, m, $C_{12}H$), 3.28 (2H, d, $^3J_{H\text{-}H}$ 6.6, $C_{11}H_2$), 7.18 - 8.04 (8H, m, Ar*H*), 8.56 (1H, d of d, $^3J_{HH}$ 4.8, $^4J_{HH}$ 1.5, ArC_1H), Minor isomer: 0.93 (6H, d, $^3J_{H\text{-}H}$ 6.6, $C_{13}H_3$ + $C_{14}H_3$), 1.98 - 2.16 (1H, m, $C_{12}H$), 3.19 (2H, d, $^3J_{H\text{-}H}$ 6.9, $C_{11}H_2$), 7.15 - 8.07 (8H, m, Ar*H*), 8.75 (1H, d of d, $^3J_{HH}$ 4.8, $^4J_{HH}$ 1.8, ArC_1H), δ_C (75 MHz, $CDCl_3$) Major isomer: 20.9 (C_{13} + C_{14}), 30.1 (C_{12}), 61.8 (C_{11}), 122.4 (ArC_4), 123.8 (ArC_2), 128.0 (ArC_9), 128.2 (ArC_8), 128.3 (ArC_{10}), 136.2 (ArC_3H), 136.5 (ArC_7), 149.0 (ArC_1H), 157.7 (ArC_5), 167.9 (*C*=N), Minor isomer: 20.8 (C_{13} + C_{14}), 30.2 (C_{12}), 61.4 (C_{11}),

123.0 (ArC_2/ArC_4), 123.4 (ArC_2/ArC_4), 128.1 (ArC_8 + ArC_9), 129.8 (ArC_{10}), 136.1 (ArC_3H), 139.1 (ArC_7), 150.0 (ArC_1H), 156.1 (ArC_5), 165.8 (C=N); *m/z* (ESI) 239 (M+1, 100 %), 240 (M+2, 39 %); HRMS calcd. for $C_{16}H_{18}N_2$ 239.1548, found 239.1541.

Synthesis of 3

A mixture of palladium(II) chloride-1,5-cyclooctadiene (0.10 g, 0.35 mmol), *iso*-butyl-(phenyl-pyridin-2-yl-methylene)-amine (0.09 g, 0.38 mmol) and 10 mL of CH_2Cl_2 was stoppered and stirred for 6 h. No precipitate formed so the reaction mixture was heated at reflux overnight. The product was concentrated to a bright orange solid under reduced pressure. It was then dissolved in the minimum amount of CH_2Cl_2 and layered with hexane. It was left overnight and the solid precipitated out. This was collected and washed with hexane (3 x 5 mL) to give the desired compound as bright orange crystals (0.12 g, 86%); ν_{max} (KBr)/cm^{-1} 1586 (C=N); δ_H (400 MHz; $CDCl_3$) 0.80 (6H, d, $^3J_{H-H}$ 6.8, $C_{13}H_3$ + $C_{14}H_3$), 2.71 (1H, septet, $^3J_{H-H}$ 6.8, $C_{12}H$), 3.66 (2H, d, $^3J_{H-H}$ 6.4, $C_{11}H_2$), 7.12 (1H, d of d, $^3J_{HH}$ 8.0 , $^4J_{HH}$ 0.8, ArC_{10}H), 7.34 - 7.36 (2H, m, Ar*H*), 7.59 - 7.67 (4H, m, Ar*H*), 7.98 (1H, t of d, $^3J_{HH}$ 8.0 , $^4J_{HH}$ 1.6, ArC_4H), 9.46 (1H, d of d, $^3J_{HH}$ 5.6 , $^4J_{HH}$ 1.2, ArC_1H). Found C, 46.09; H, 4.31; N, 6.67; Cl, 16.87; $C_{16}H_{18}N_2Cl_2Pd$ requires C, 46.23; H, 4.36; N, 6.74; Cl, 17.06.

Synthesis of precursor to 4

A mixture of *n*-pentylamine (1.19 g, 1.58 mL, 13.65 mmol), 2-benzoylpyridine (0.50 g, 2.73 mmol), titanium(IV) ethoxide (2.86 mL, 3.11 g, 13.65 mmol) and 10 mL of dichloromethane was refluxed overnight with stirring. The reaction was cooled to room temperature and deionised water (5 mL) was added slowly. The solution was filtered through celite to remove traces of solid material, and washed with CH_2Cl_2 (3 x 5 mL). The organic layer was separated and the aqueous layer was extracted with CH_2Cl_2 (3 x 5 mL). The combined organic layers were washed with brine (5 mL), dried ($MgSO_4$), filtered and concentrated to yield a colourless oil (0.57 g, 83 %). ^{1}H and ^{13}C NMR analysis showed that the reaction had gone to completion and the pure imine was obtained as E/Z isomers in a ratio of 1.0:1.4; ν_{max} (NaCl)/cm^{-1} 1629 (C=N), 1583 (C=N, pyridine ring); δ_H(300 MHz; $CDCl_3$) Major isomer: 0.85 - 0.89 (3H, m, $C_{15}H_3$), 1.20 - 1.36 (4H, m, $C_{13}H_2$ + $C_{14}H_2$), 1.66 - 1.77 (2H, m, $C_{12}H_2$), 3.45 (2H, t, $^3J_{H-H}$ 7.2, $C_{11}H_2$), 7.19 - 7.93 (8H, m, Ar*H*), 8.58 (1H, d of d, $^3J_{HH}$ 4.8 , $^4J_{HH}$ 1.5, ArC_1H), Minor isomer: 0.85 - 0.89 (3H, m, $C_{15}H_3$), 1.20 - 1.36 (4H, m, $C_{13}H_3$ + $C_{14}H_3$), 1.66 - 1.77 (2H, m, $C_{12}H_2$), 3.36 (2H, t, $^3J_{H-H}$ 7.2, $C_{11}H_2$), 7.16 - 7.96 (8H, m, Ar*H*), 8.75 (1H, d of d, $^3J_{HH}$ 4.8, $^4J_{HH}$ 1.5, ArC_1H), δ_C (75 MHz, $CDCl_3$) Major isomer: 14.0 (C_{15}), 22.5 (C_{14}), 29.8 (C_{13}), 30.8 (C_{12}), 54.2 (C_{11}), 122.6 (ArC_4), 123.8 (ArC_2), 127.9 (ArC_9), 128.2 (ArC_8), 128.3 (ArC_{10}), 136.2 (ArC_3H), 136.4 (ArC_7), 149.1 (ArC_1H), 157.6 (ArC_5), 167.9 (C=N), Minor isomer: 14.0 (C_{15}), 22.5 (C_{14}), 29.7 (C_{13}), 30.8 (C_{12}), 53.7 (C_{11}), 123.0 (ArC_4), 123.3 (ArC_2), 128.0 (ArC_8/ArC_9), 128.1 (ArC_8/ArC_9), 129.9 (ArC_{10}), 136.1 (ArC_3H), 139.0 (ArC_7), 150.0 (ArC_1H), 156.1 (ArC_5), 165.9 (C=N); *m/z* (ESI) 253 (M+1, 100 %), 254 (M+2, 31 %), 255 (M+3, 3 %); HRMS calcd. for $C_{17}H_{20}N_2$ 253.1705, found 253.1712.

Synthesis of 4

A mixture of palladium(II) chloride-1,5-cyclooctadiene[1] (0.10 g, 0.35 mmol), pentyl-(phenyl-pyridin-2-yl-methylene)-amine (0.09 g, 0.36 mmol) and 10 mL of dichloromethane was stoppered and stirred for 6 h. No precipitate formed so the reaction mixture was heated at reflux overnight. The product was concentrated to a bright orange solid under reduced pressure. It was then dissolved in the minimum amount of CH_2Cl_2 and layered with hexane. It was left overnight and the solid precipitated out. This was collected and washed with hexane (3 x 5 mL) to give the desired compound as a bright orange crystalline solid (0.11 g, 79%); ν_{max} (KBr)/cm^{-1} 1588 (C=N); δ_H(400 MHz; $CDCl_3$) 0.78 (3H, t, $^3J_{H-H}$ 6.8 $C_{15}H_3$), 1.12 - 1.18 (4H, m, $C_{13}H_2 + C_{14}H_2$), 1.77 - 1.84 (2H, m, $C_{12}H_2$), 3.73 (2H, t, $^3J_{H-H}$ 7.6, $C_{11}H_2$), 7.07 (1H, d of d, $^3J_{HH}$ 8.0 , $^4J_{HH}$ 0.8, ArC$_{10}$H), 7.43 -7.45 (2H, m, Ar*H*), 7.59 - 7.65 (4H, m, Ar*H*), 7.95 (1H, t of d, $^3J_{HH}$ 7.6 , $^4J_{HH}$ 1.6, ArC$_4$H), 9.39 (1H, d of d, $^3J_{HH}$ 5.6 , $^4J_{HH}$ 1.2, ArC$_1$H). Found C, 47.37; H, 4.49; N, 6.42; Cl, 16.20; $C_{17}H_{20}N_2Cl_2Pd$ requires C, 47.52; H, 4.69; N, 6.52; Cl, 16.50.

Synthesis of 11

4-Nitrobenzene thiol (1.71g, 11.0 mmol) was added dropwise to a stirred suspension of NaH (0.46g, 60 % dispersion in oil, 11.6 mmol) in DMF (15 mL) under N_2 at 0 °C. The reaction was stirred for 20 minutes and 4-iodobenzyl bromide (3.27g, 11.0 mmol) in DMF (1 mL) was then slowly added over 20 minutes. The reaction mixture was stirred for a further 16 h at room temperature. Water (50 mL) and CH_2Cl_2 (30 mL) were added and the phases separated. The organic layer was concentrated under reduced pressure. The product was dissolved in CH_2Cl_2 (30 mL), washed with 2M HCl (3 x 20 mL) and brine (1 x 20 mL), dried over $MgSO_4$ and concentrated under reduced pressure to give the crude product. Purification by recrystallisation from hot methanol afforded the title compound as a yellow solid (3.62g, 91 %), mp 138-140 ^oC; ν_{max} (KBr)/cm^{-1} 1594, 1578, 1506 (asymmetric NO_2 stretch), 1331 (symmetric NO_2 stretch), 475 (C-I stretch); δ_H (300 MHz) ($CDCl_3$) 4.18 (2H, s, CH_2), 7.08-7.17 (2H, m, ArCH), 7.28-7.36 (2H, m, ArH), 7.62-7.70 (2H, m, ArH), 8.06-8.15 (2H, m, ArH); δ_c (75.5 MHz) ($CDCl_3$) 36.6 (CH_2, **C**H_2), 93.3 (C, aromatic **C**-I), 124.0, 126.9, 130.6 (CH, 3 x aromatic **C**H), 135.3 (C, aromatic **C**), 138.0 (CH, aromatic **C**H), 146.5 (C, aromatic **C**), 1 x aromatic **C** signal absent; m/z (ESI) 326.2 [$(M-NO_2)^+$].

Synthesis of 15

To a solution of triethylamine (2 mL, 1.45 g, 14.35 mmol) and sulfathiazole (3.99 g, 15.64 mmol) in dry methanol (26 mL), was slowly added di-tert-butyldicarbonate (4.12 g, 18.90 mmol) in dry methanol (1 mL) at 0-5 °C. The resulting suspension was stirred at room temperature for 24 h. TLC analysis (CH_2Cl_2: methanol, 90:10) showed product and starting material and the solution was pH = 8, so additional di-tert-butyl dicarbonate (1.35 g, 6.21 mmol) in methanol (3 mL) was added along with triethylamine (0.9 mL) and the reaction stirred at room temperature for 3 h. The reaction mixture was separated between HCl (2 M, 30 mL) and ethyl acetate (30 mL). The aqueous layer was separated and extracted with ethyl acetate (3 x 20 mL), washed with brine (10 mL) and the combined organic extracts were

dried and concentrated to give a crude yellow solid, which was immediately recrystallised twice from cold methanol/water to give a white solid (0.685 g, 1.93 mmol, 12%), mp 188-190 ºC. δ_H (300MHz; DMSO-d_6) 1.48 (9H, s, C(C*H*$_3$)$_3$, 6.79-6.80 (1H, d, ^{3}J=4.62 Hz , CHC*H*), 7.22-7.23 (1H, d, ^{3}J =4.65 Hz , C*H*CH), 7.55-7.58 (2H, d, ^{3}J=8.82 Hz , Ar-*H*), 7.67-7.69 (2H, d, ^{3}J =8.79 Hz, Ar-*H*), 9.72 (1H, br s, N*H*); δ_C (75 MHz; DMSO-d_6) 26.88 (-*C*H$_3$), 78.58 (*C*-CH$_3$), 106.84, 124.44 (-*C*H), 116.29 (2 x Ar-*C*H), 125.77 (2 x Ar-*C*H), 134.09, 141.72 (Quaternary Ar-*C*), 151.39 (*C*=O); υ_{max}(KBr/cm^{-1}) 3345 (N-H), 3117 (ArylC-H), 1718 (C=O), 1593(C=C), 1330, 1147(SO_2); Found: C, 47.23; H, 4.87; N, 12.02; $C_{14}H_{17}N_3O_4S_2$ requires C, 47.31; H, 4.82; N, 11.82.

Crystallisation of 16

Sulfaguanidine (0.214 g, 1.000 mmol) was dissolved in bulk acetone (40 mL) and allowed to stand at ambient temperature over 2 days to afford white needle crystals (0.196 g, 92%) suitable for single X-ray diffraction.

Synthesis of 17

Triphenylphosphine oxide (0.278 g, 1.000 mmol) and thiobenzamide (0.137 g, 1.000 mmol) were dissolved in acetone (20 mL). The solution was allowed to stand at ambient temperature over 3 days to afford a yellow solid (0.336 g, 81%), mp 129 – 131 °C. Found: C, 71.95; H, 3.58; N, 5.40; S, 8.20; $C_{25}H_{22}NOPS$ requires C, 72.27; H, 3.37; N, 5.34; S, 7.72.

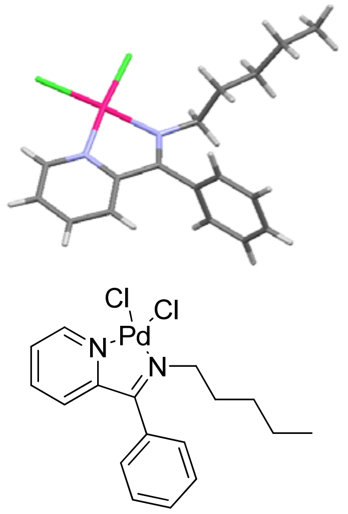

Compound **3**

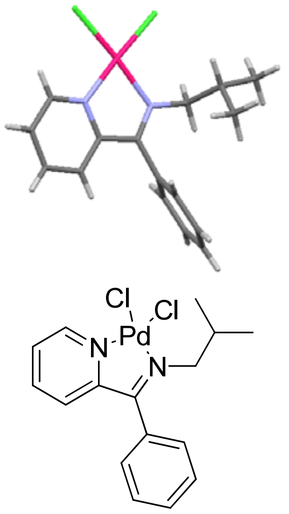

Compound **4**

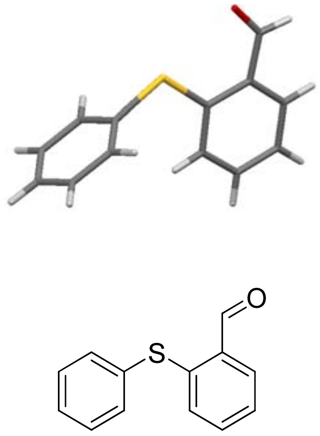

Compound **5**

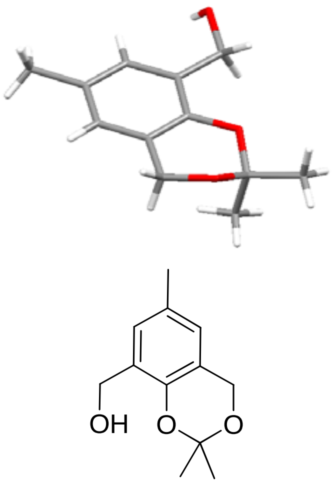

Compound **6**

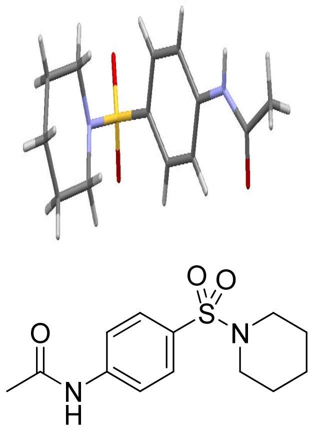

Compound **7**

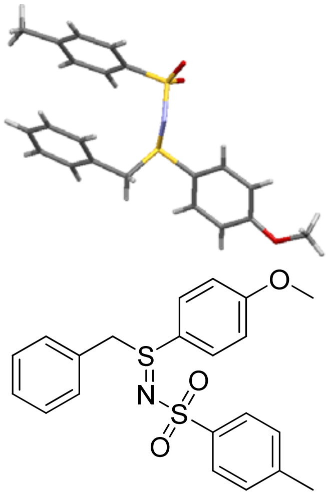

Compound **8**

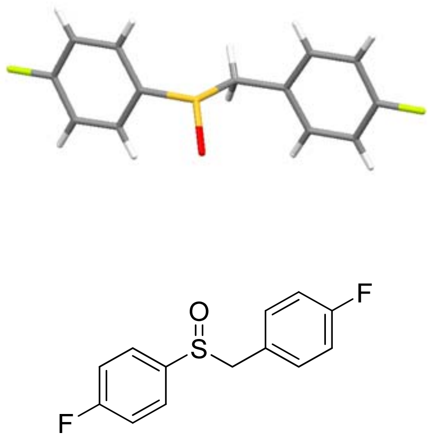

Compound **9**

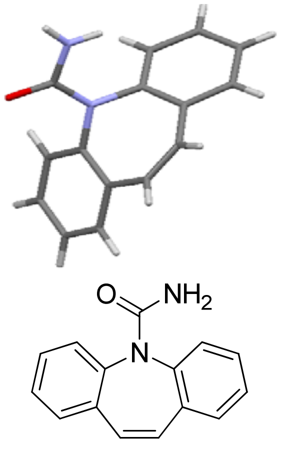

Compound **10**

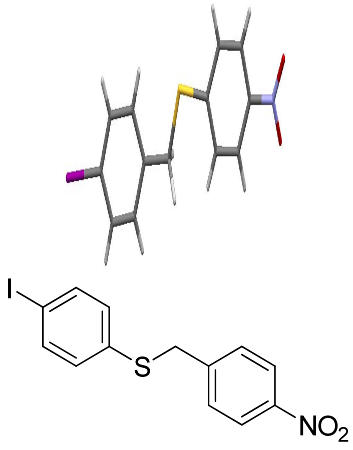

Compound **11**

Scheme 1: Chemical representation and molecular representation based on data obtained from the Autostructure™ software for compounds **3–11**.

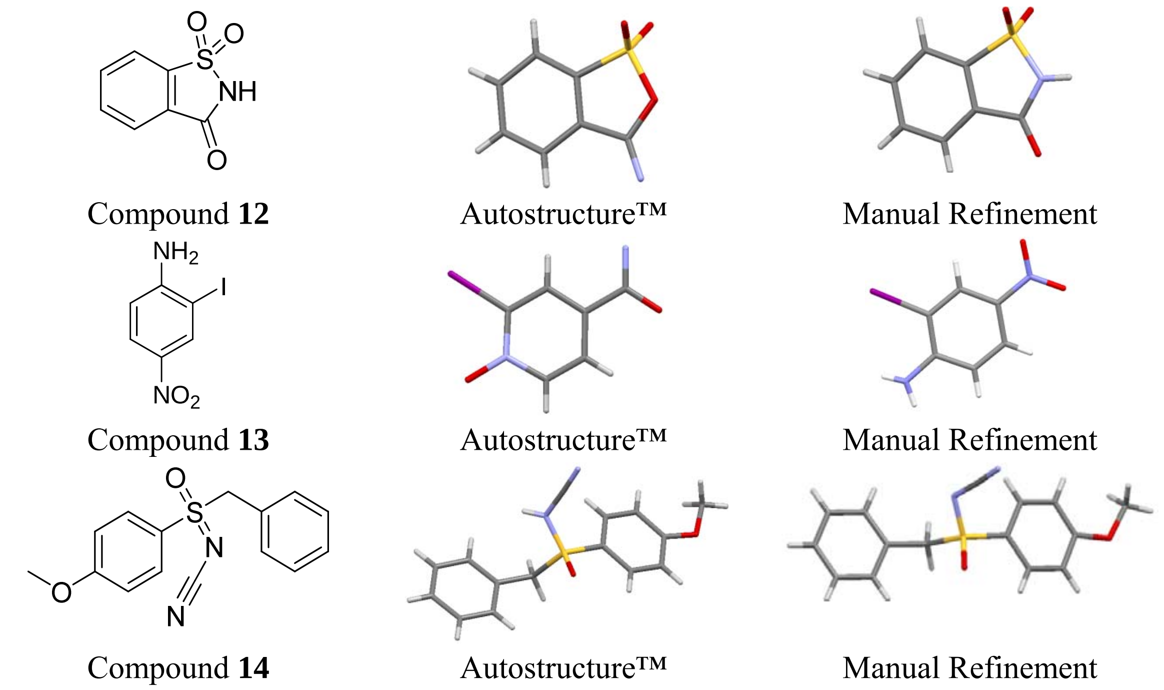

Scheme 2: Overview of the incorrect structures, compounds **12 – 14**. For clarity, only one of the unique molecules in **13** is shown.

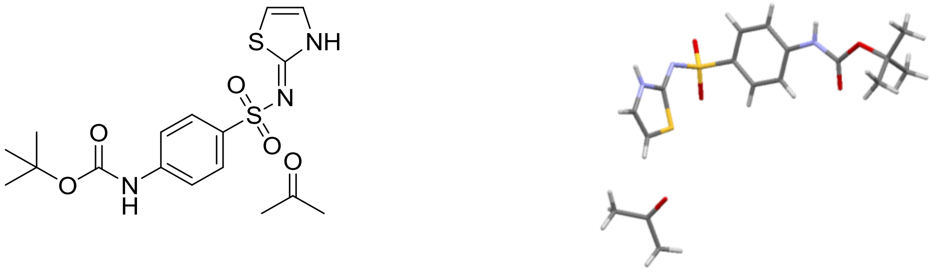

Compound **15**

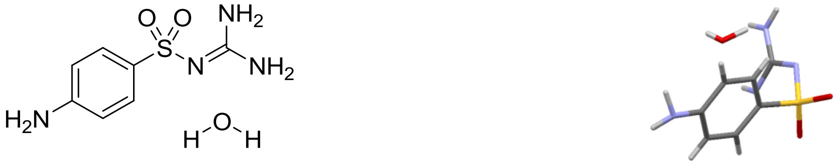

Compound **16**

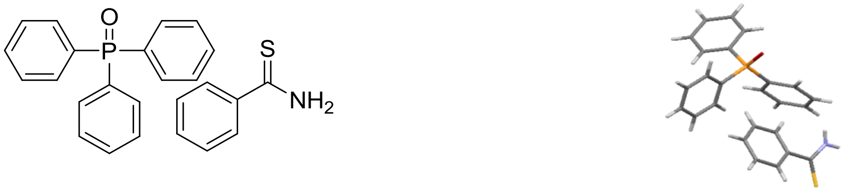

Compound **17**

Scheme 3: Multi-component systems for compounds **15–17.**

Table 1: Crystallographic Data for Compound **1**.

Position of crystal	Centre	Below	Side
Exposure time per frame, s	10	60	60
Resolution, Å	0.84	0.92	0.84
Unit-cell dimensions, Å & °	a = 24.003(3)<br>b = 4.7435(6)<br>c = 10.8255(12)<br><br>β = 90.185(4)	a = 4.736(3)<br>b = 10.819(5)<br>c = 12.206(6)<br>α = 89.727(15)<br>β = 78.904(14)<br>γ = 89.977(18)	a = 24.012(3)<br>b = 4.7429(5)<br>c = 10.8321(13)<br><br>β = 90.197(4)
V, Å^3	1232.6(3)	613.6(5)	1233.6(3)
Total reflections	3757	4509	3662
Unique reflections	1094	1596	1097
Observed reflections, $I > 2\sigma(I)$	894	628	960
R_{int}	0.031	0.200	0.024
GooF	1.16	0.98	1.20
$R1$, $I > 2\sigma(I)$	0. 036	0.099	0.038
$wR2$, all data	0.132	0.321	0.122
$\Delta\rho_{max}$, $\Delta\rho_{min}$	0.24, -0.33	0.42, -0.42	0.24, -0.33
Mean C-C Bond Precision, Å	0.0037	0.0197	0.0033

Table 2: Crystallographic data obtained by varying the crystal size of compound **2**.

	Sample 1	Sample 2	Sample 3	Sample 4	Sample 5
Crystal Shape	Block	Block	Block	Block	Block
Crystal size, mm	0.21 x 0.35 x 0.50	0.15 x 0.34 x 0.39	0.14 x 0.21 x 0.27	0.13 x 0.20 x 0.23	0.12 x 0.16 x 0.18
Exposure time per frame, s	5	60	60	60	Failed
Unit-cell dimensions, Å & °	a = 10.1414(9)<br>b = 11.3865(9)<br>c = 11.3289(9)<br>β = 97.400(3)	a = 10.1385(11)<br>b = 11.3898(11)<br>c = 11.3325(12)<br>β = 97.467(4)	a = 10.1375(11)<br>b = 11.3871(14)<br>c = 11.3412(14)<br>β = 97.482(4)	a = 10.137 (3)<br>b = 11.413 (4)<br>c = 11.345(3)<br>β = 97.465(11)	-
V, $Å^3$	1297.31(19)	1297.5(2)	1298.0(3)	1301.5(7)	-
Total reflections	8247	12421	8248	8133	-
Unique reflections	2277	2291	2275	2277	-
Observed reflections, $I > 2\sigma(I)$	1729	1818	1692	1626	-
R_{int}	0.029	0.041	0.043	0.043	-
GooF	1.08	1.09	1.09	1.09	-
R_1, $I > 2\sigma(I)$	0.038	0.035	0.038	0.040	-
wR_2, all data	0.147	0.115	0.122	0.125	-
$\Delta\rho_{max}$, $\Delta\rho_{min}$	0.27, -0.35	0.258, -0.209	0.233, -0.229	0.215, -0.181	-
Mean C-C Bond Precision, Å	0.0040	0.0038	0.0039	0.0047	-

Table 3:Crystallographic data obtained for compounds **3 – 11**.

	Compound **3**	Compound **4**	Compound **5**	Compound **6**	Compound **7**
Empirical Formula	$C_{17}H_{20}Cl_2N_2Pd$	$C_{16}H_{18}Cl_2N_2Pd$	$C_{13}H_{10}OS$	$C_{12}H_{16}O_3$	$C_{13}H_{18}N_2O_3S$
Crystal Shape	Plate	Block	Block	Block	Plate
Crystal size, mm	0.29 x 0.31 x 0.35	0.19 x 0.31 x 0.51	0.27 x 0.31 x 0.31	0.27 x 0.38 x 0.44	0.26 x 0.27 x 0.31
Exposure time per frame, s	5	5	10	10	10
Crystal System	Monoclinic	Triclinic	Monoclinic	Monoclinic	Monoclinic
Space Group, Z	$P2_1/c$, 4	$P\bar{1}$, 2	$P2_1/n$, 4	$P2_1/c$, 4	$P2_1/c$, 4
Unit-cell dimensions, Å & °	a = 9.356(2)<br>b = 9.9295(19)<br>c = 19.357(4)<br>β = 90.470(7)	a = 8.5305(11)<br>b = 9.6211(14)<br>c = 10.9880(16)<br>α = 72.593(4)<br>β = 78.780(5)<br>γ = 83.039(4)	a = 8.007(2)<br>b = 16.958(5)<br>c = 8.031(2)<br>β = 95.097(10)	a = 8.894(4)<br>b = 16.220(8)<br>c = 7.742(3)<br>β = 90.214(14)	a = 12.8651(14)<br>b = 15.3291(14)<br>c = 7.1435(8)<br>β = 93.379(4)
V, $Å^3$	1798.2(7)	842.2(2)	1086.1(5)	1116.9(9)	1406.3(3)
Total reflections	16537	8425	6685	7217	13591
Unique reflections	3229	3017	1910	1944	2482
Observed reflections, $I > 2\sigma(I)$	2821	2740	1366	1285	1862
R_{int}	0.066	0.025	0.049	0.043	0.049
GooF	1.15	1.23	1.07	1.06	0.97
R_1, $I > 2\sigma(I)$	0.039	0.022	0.047	0.046	0.040
wR_2, all data	0.120	0.091	0.153	0.165	0.138
$\Delta\rho_{max}$, $\Delta\rho_{min}$	0.76, -1.66	0.66, -0.78	0.32, -0.26	0.31, -0.33,	0.31, -0.37
Mean C-C Bond	0.0067	0.0057	0.0045	0.0032	0.0037

Precision, Å					

	Compound **8**	Compound **9**	Compound **10**	Compound **11**
Empirical Formula	$C_{21}H_{22}NO_3S_2$	$C_{13}H_{10}F_2OS$	$C_{15}H_{12}N_2O$	$C_{13}H_{10}INO_2S$
Crystal Shape	Block	Block	Block	Plate
Crystal size, mm	0.27 x 0.41 x 0.43	0.24 x 0.27 x 0.31	0.23 x 0.27 x 0.31	0.31 x 0.27 x 0.11
Exposure time per frame, s	5	60	5	5
Crystal System	Monoclinic	Orthorhombic	Monoclinic	Triclinic
Space Group, Z	$P2_1/c$, 4	$Pca2_1$, 4	$P2_1/n$, 4	$P\bar{1}$, 2
Unit-cell dimensions, Å & °	a = 11.7937(9)<br>b = 11.4816(7)<br>c = 14.9358(12)<br>β = 95.972(3)	a = 8.3649(16)<br>b = 5.5063(10)<br>c = 25.129(4)	a = 7.5500(16)<br>b = 11.186(3)<br>c = 13.954(3)<br>β = 92.938(8)	a = 7.5076(15)<br>b = 7.760(2)<br>c = 11.507(3)<br>α = 95.317(8)<br>β = 103.999(7)<br>γ = 97.013(3)
V, $Å^3$	2011.5(3)	1157.4(4)	1176.9(5)	640.4(3)
Total reflections	12920	6646	7461	6315
Unique reflections	3546	2013	2066	2224
Observed reflections, $I > 2\sigma(I)$	2664	1760	1550	1836
R_{int}	0.035	0.037	0.0332	0.038
GooF	1.10	1.19	1.22	1.07
R_1, $I > 2\sigma(I)$	0.043	0.034	0.040	0.030
wR_2, all data	0.143	0.118	0.125	0.097

$\Delta\rho_{max}$, $\Delta\rho_{min}$	0.34, -0.33	0.18, -0.19	0.14, -0.20	1.07, -0.55
Mean C-C Bond Precision, Å	0.0036	0.0055	0.0027	0.0080
Flack	-	-0.07(10)	-	-

Table 4: Crystallography data obtained for compounds **12 – 14.**

	Compound **12**	Compound **13**	Compound **14**
Empirical Formula	$C_7H_5NO_3S$	$C_6H_5IN_2O_2$	$C_{15}H_{14}N_2O_2S_1$
Crystal Shape	Plate	Block	Block
Crystal size, mm	0.11 x 0.25 x 0.32	0.40 x 0.45 x 0.45	0.21 x 0.31 x 0.33
Exposure time per frame, s	10	5	10
Crystal System	Monoclinic	Monoclinic	Orthorhombic
Space Group, *Z*	$P2_1/c$, 4	$P2_1/c$, 8	$P2_12_12_1$, 4
Unit-cell dimensions, Å & ^{o}	*a* = 9.6083(16)<br>*b* = 6.9347(10)<br>*c* = 11.882(2)<br>*β* = 103.834(6)	*a* = 7.7617(15)<br>*b* = 12.912(3)<br>*c* = 15.663(3)<br>*β* = 95.141(6)	*a* = 7.7592(10)<br>*b* = 9.9677(13)<br>*c* = 18.230(2)
V, $Å^3$	768.7(2)	1563.5(6)	1409.9(3)
Total reflections	4799	9857	8816
Unique reflections	1346	2799	2458
Obs. reflns, $I > 2\sigma(I)$	1025	2259	1984
R_{int}	0.035	0.030	0.041
Autostructure™			
GooF	1.08	1.04	1.14
$R1$, $I > 2\sigma(I)$	0.059	0.033	0.040
$wR2$, all data	0.197	0.104	0.113
$\Delta\rho_{max}$, $\Delta\rho_{min}$	0.59, -0.47	0.96, -0.76	0.15, -0.27
Flack	-	-	0.43(12)
Mean C-C Bond Precision, Å	0.0061	0.0087	0.0047
Manual Refinement			
GooF	1.17	1.02	1.05
$R1$, $I > 2\sigma(I)$	0.039	0.026	0.037
$wR2$, all data	0.138	0.056	0.081
$\Delta\rho_{max}$, $\Delta\rho_{min}$	0.24 , -0.31	0.83, -0.65	0.18, -0.20
Flack	-	-	0.43 (8)
Mean C-C Bond Precision, Å	0.0039	0.0050	0.0037

Table 5: Crystallographic data obtained for compound **15 – 17**.

	Compound **15**	Compound **16**	Compound **17**
Empirical Formula	$C_{17}H_{23}N_3O_5S_2$	$C_7H_{11}N_4O_3S$	$C_{25}H_{22}NOPS$
Crystal Shape	Block	Plate	Plate
Crystal size, mm	0.20 x 0.30 x 0.50	0.12 x 0.12 x 0.32	0.10 x 0.21 x 0.45
Exposure time per frame, s	5	10	60
Crystal System	Monoclinic	Monoclinic	Triclinic
Space Group, Z	$P2_1/n$, 4	$P2_1$, 2	$P\bar{1}$, 2
Unit-cell dimensions, Å & ^{o}	a = 7.811(4)<br>b = 17.503(10)<br>c = 15.892(9)<br><br>β = 103.305(17)	a = 5.6245(17)<br>b = 7.354(3)<br>c = 12.521(5)<br><br>β = 93.445(13)	a = 10.2699(10)<br>b = 11.1605(10)<br>c = 11.1911(12)<br>α = 85.945(3)<br>β = 67.750(3)<br>γ = 70.671(3)
V, Å^3	2114(2)	517.0(3)	1118.07(19)
Total reflections	19952	3381	11064
Unique reflections	3694	1776	3902
Observed reflections, $I > 2\sigma(I)$	2806	1427	3000
R_{int}	0.044	0.039	0.035
GooF	1.12	1.00	1.01
$R1$, $I > 2\sigma(I)$	0.045	0.046	0.040
$wR2$, all data	0.142	0.092	0.146
Flack	-	-0.25(12)	-
$\Delta\rho_{max}$, $\Delta\rho_{min}$	0.36, -0.28	0.24, -0.28	0.27, -0.29
Mean C-C Bond Precision, Å	0.0041	0.0050	0.0043

Table 6: Comparison of crystallography data obtained from the SMART X2S and an APEX DUO for compounds **2**, **8**, **12** and **16**.

	Compound **2** SMART X2S	Compound **2** APEX DUO	Compound **8** SMART X2S	Compound **8** APEX DUO
Unit-cell dimensions, Å & º	*a* = 10.1375(11)<br>*b* = 11.3871(14)<br>*c* = 11.3412(14)<br>*β* = 97.482(4)	*a* = 10.100(2)<br>*b* = 11.365(2)<br>*c* = 11.300(2)<br>*β* = 97.464(5)	*a* = 11.7937(9)<br>*b* = 11.4816(7)<br>*c* = 14.9358(11)<br>*β* = 95.972(3)	*a* = 11.7771(7)<br>*b* = 11.4544(6)<br>*c* = 14.9031(9)<br>*β* = 95.9450(10)
V, Å^3	1298.0(3)	1286.1(4)	2011.5(3)	1999.6(2)
Detector distance, mm	39	37	39	40
Exposure time per frame, s	60	10	5	5
Resolution, Å	0.84	0.85	0.84	0.84
Total time, h	11.57	5.20	2.06	5.10
Total reflections	8248	13491	12920	26025
Unique reflections	2275	2180	3546	3551
Observed reflections, $I > 2\sigma(I)$	1692	1698	2664	3109
R_{int}	0.043	0.039	0.035	0.024
GooF	1.09	1.03	1.10	1.03
$R1$, $I > 2\sigma(I)$	0.038	0.033	0.043	0.031
$wR2$, all data	0.122	0.085	0.143	0.085
$\Delta\rho_{max}$, $\Delta\rho_{min}$	0.23, -0.23	0.14, -0.27	0.34, -0.33	0.30, -0.27
Mean C-C Bond Precision, Å	0.0039	0.0032	0.0036	0.0025

	Compound **12** SMART X2S	Compound **12** APEX DUO	Compound **16** SMART X2S	Compound **16** APEX DUO
Unit-cell dimensions, Å & º	*a* =9.6083(16)<br>*b* = 6.9347(10)<br>*c* = 11.882(2)<br>*β* = 103.834(6)	*a* = 9.5830(12)<br>*b* = 6.9252(8)<br>*c* = 11.8518(15)<br>*β* = 103.815(3)	*a* = 5.6245(17)<br>*b* = 7.354(3)<br>*c* = 12.521(5)<br>*β* = 93.445(13)	*a* = 5.6095(10)<br>*b* = 7.3299(15)<br>*c* = 12.491(2)<br>*β* = 93.297(5)
V, Å^3	768.7(2)	763.78(16)	517.0(3)	512.74(17)
Detector distance, mm	39	38	39	38
Exposure time per frame, s	5	5	10	10
Resolution, Å	0.84	0.82	0.84	0.84
Total time, h	2.06	2.33	3.40	3.27

Total reflections	4799	5166	3381	3017
Unique reflections	1346	1473	1776	1940
Observed reflections, $I > 2\sigma(I)$	1025	1283	1427	1825
R_{int}	0.035	0.022	0.039	0.019
GooF	1.17	1.06	1.00	1.13
$R1$, $I > 2\sigma(I)$	0.039	0.031	0.046	0.031
$wR2$, all data	0.138	0.081	0.092	0.073
$\Delta\rho_{max}$, $\Delta\rho_{min}$	0.24 , -0.31	0.28, -0.31	0.24, -0.28	0.21, -0.22
Mean C-C Bond Precision, Å	0.0039	0.0026	0.0050	0.0030
Flack	-	-	-0.25(12)	-0.02(8)

Table 7: Comparison of crystallography data obtained from the SMART X2S and an APEX DUO for compounds **11** and **18** (4-methyl-*N*-phenyl-benzenesulfonamide).

	Compound **11** SMART X2S	Compound **11** APEX DUO	Compound **18** SMART X2S	Compound **18** APEX DUO
Empirical Formula	$C_{13}H_{10}INO_2S$	$C_{13}H_{10}INO_2S$	$C_{13}H_{12}NO_2S$	$C_{13}H_{12}NO_2S$
Crystal size, mm	0.11 x 0.17 x 0.31	0.11 x 0.17 x 0.31	0.18 x 0.30 x 0.32	0.18 x 0.30 x 0.32
Exposure time per frame, s	5	5	60	10
Detector Distance	39	37	39	37
Unit-cell dimensions, Å & º	*a* = 7.5076(15)<br>*b* = 7.760(2)<br>*c* = 11.507(3)<br>*α* = 95.317(8)<br>*β* = 103.999(7)<br>*γ* = 97.013(3)	*a* = 7.4946(11)<br>*b* = 7.7421(11)<br>*c* = 11.4816(16)<br>*α* = 95.418(3)<br>*β* = 103.879(3)<br>*γ* = 96.985(3)	*a* = 10.2890(11)<br>*b* = 11.0563(12)<br>*c* = 13.4402(15)<br>*α* = 67.823(3)<br>*β* = 87.573(4)<br>*γ* = 67.616(3)	*a* = 10.259(3)<br>*b* = 11.050(4)<br>*c* = 13.380(5)<br>*α* = 67.808(8)<br>*β* = 87.291(8)<br>*γ* = 67.435(7)
V, $Å^3$	640.4(3)	636.65(16)	1299.9(2)	1288.4(7)
Total reflections	6315	7653	12855	23008
Unique reflections	2224	2786	4545	4600
Obs. reflections, $I > 2\sigma(I)$	1836	2492	3364	3117
R_{int}	0.038	0.025	0.036	0.039
GooF	1.07	1.05	1.01	1.10
$R1$, $I > 2\sigma(I)$	0.030	0.028	0.055	0.062
wR_2, all data	0.097	0.066	0.174	0.266
$\Delta\rho_{max}$, $\Delta\rho_{min}$	1.07, -0.55	1.41, -0.43	0.25, -0.40	0.37, -0.44
Mean C-C Bond Precision, Å	0.0055	0.0045	0.0059	0.0079
